# Association Between Push-up Exercise Capacity and Future Cardiovascular Events Among Active Adult Men

**DOI:** 10.1001/jamanetworkopen.2018.8341

**Published:** 2019-02-15

**Authors:** Justin Yang, Costas A. Christophi, Andrea Farioli, Dorothee M. Baur, Steven Moffatt, Terrell W. Zollinger, Stefanos N. Kales

**Affiliations:** 1 Harvard T.H. Chan School of Public Health, Boston, Massachusetts; 2 Cambridge Health Alliance, Harvard Medical School, Cambridge, Massachusetts; 3 Cyprus International Institute for Environmental and Public Health, Cyprus University of Technology, Limassol, Cyprus; 4 Department of Medical and Surgical Sciences (DIMEC), University of Bologna, Bologna, Italy; 5 Public Safety Medical, Indianapolis, Indiana; 6 Department of Epidemiology, Richard M. Fairbanks School of Public Health, Indiana University, Indianapolis

## Abstract

**Importance:**

Cardiovascular disease (CVD) remains the leading cause of mortality worldwide. Robust evidence indicates an association of increased physical fitness with a lower risk of CVD events and improved longevity; however, few have studied simple, low-cost measures of functional status.

**Objective:**

To evaluate the association between push-up capacity and subsequent CVD event incidence in a cohort of active adult men.

**Design, Setting, and Participants:**

Retrospective longitudinal cohort study conducted between January 1, 2000, and December 31, 2010, in 1 outpatient clinics in Indiana of male firefighters aged 18 years or older. Baseline and periodic physical examinations, including tests of push-up capacity and exercise tolerance, were performed between February 2, 2000, and November 12, 2007. Participants were stratified into 5 groups based on number of push-ups completed and were followed up for 10 years. Final statistical analyses were completed on August 11, 2018.

**Main Outcomes and Measures:**

Cardiovascular disease–related outcomes through 2010 included incident diagnoses of coronary artery disease and other major CVD events. Incidence rate ratios (IRRs) were computed, and logistic regression models were used to model the time to each outcome from baseline, adjusting for age and body mass index (BMI) (calculated as weight in kilograms divided by height in meters squared). Kaplan-Meier estimates for cumulative risk were computed for the push-up categories.

**Results:**

A total of 1562 participants underwent baseline examination, and 1104 with available push-up data were included in the final analyses. Mean (SD) age of the cohort at baseline was 39.6 (9.2) years, and mean (SD) BMI was 28.7 (4.3). During the 10-year follow up, 37 CVD-related outcomes (8601 person-years) were reported in participants with available push-up data. Significant negative associations were found between increasing push-up capacity and CVD events. Participants able to complete more than 40 push-ups were associated with a significantly lower risk of incident CVD event risk compared with those completing fewer than 10 push-ups (IRR, 0.04; 95% CI, 0.01-0.36).

**Conclusions and Relevance:**

The findings suggest that higher baseline push-up capacity is associated with a lower incidence of CVD events. Although larger studies in more diverse cohorts are needed, push-up capacity may be a simple, no-cost measure to estimate functional status.

## Key Points

**Question:**

Is there an office-based objective measurement that clinicians can use to assess the association between fitness and cardiovascular disease risk?

**Findings:**

This longitudinal cohort study of 1104 occupationally active adult men found a significant negative association between baseline push-up capacity and incident cardiovascular disease risk across 10 years of follow-up. Participants able to complete more than 40 push-ups were associated with a significant reduction in incident cardiovascular disease event risk compared with those completing fewer than 10 push-ups.

**Meaning:**

Push-up capacity is a no-cost, fast, and simple measure that may be a useful and objective clinical assessment tool for evaluating functional capacity and cardiovascular disease risk.

## Teaser

This cohort study investigates the association between participants’ capacity to complete push-ups at baseline and cardiovascular disease events during 10 years of follow-up.

## Introduction

Cardiovascular disease (CVD) remains the leading cause of death worldwide.^1^ In addition to long-recognized risk factors for CVD, such as smoking, hypertension, and diabetes, the unfavorable health consequences of physical inactivity on cardiovascular health have been well established.^2,3,4,5^ Studies have suggested that physical activity provides cardiovascular benefits independent of other modifiable CVD risk factors associated with a lower incidence of multiple diseases, including CVD, diabetes, cancer, and Alzheimer disease.^3,6,7,8,9^ A recent US study further suggested that moderate to vigorous physical activity could significantly reduce premature mortality and prolong life expectancy.^10^ Given this robust scientific evidence, the American Heart Association added physical activity to its My Life Check—Life’s Simple 7 campaign to reduce the burden of CVD and improve overall health.^11,12^

In multiple recent scientific and policy statements, the American Heart Association has promoted assessment of physical activity in clinical settings and workplaces.^2,3,13,14,15,16^ Ross et al^17^ and Golightly et al^18^ have also emphasized the growing evidence for objectively assessing cardiorespiratory fitness (CRF) as a vital sign in health care settings. However, unlike anthropometric measurements and serum biomarkers, physical activity and CRF assessments have largely been neglected by clinicians. The most commonly used physical activity assessments are the patient’s self-reported history and health and lifestyle questionnaires.^2^ However, objectively measured CRF levels are often significantly lower than expected based on self-reported physical activity.^19,20^ Although good performance on accurate and objective CRF assessment tools such as exercise tolerance tests has been inversely associated with future CVD,^21,22^ these examinations are expensive, time-consuming, and often require professional facilities and trained personnel to administer. The use of these tools remains limited to particular occupations and targeted patient populations. To our knowledge, no study has examined the association of push-up capacity, a simple, no-cost, surrogate measure of functional status, with future cardiovascular events. In this study, we examined baseline performance on commonly performed physical fitness assessments (push-up capacity and submaximal treadmill tests) and its association with subsequent incident CVD events in a cohort of occupationally active men. We hypothesized that higher fitness levels would be associated with lower rates of incident CVD.

## Methods

### Study Population

This study followed the Strengthening the Reporting of Observational Studies in Epidemiology (STROBE) reporting guideline for cohort studies.^23^ A retrospective cohort was assembled from records of active career firefighters from 10 Indiana fire departments who underwent periodic medical surveillance between January 1, 2000, and December 31, 2010, at 1 local medical clinic under contract with the fire departments. Male firefighters aged 18 years or older who had no job restrictions at the time of their initial examination were included in the study. Each underwent baseline and periodic physical examinations, including tests of push-up capacity and maximal or submaximal exercise tolerance tests between February 2, 2000, and November 12, 2007. The study protocol was reviewed by the institutional review board of the Harvard T.H. Chan School of Public Health, which also determined that the retrospective review of the firefighters’ deidentified records was exempt from further review and informed consent.

### Data Collection

Data collected during baseline and follow-up visits included complete physical examinations, anthropometric measures, and laboratory results, as well as clinical and occupational outcomes. All examinations were voluntary, and participants were allowed to bypass any part of the examination. The clinical examination was conducted by trained nurse practitioners and consisted of standardized measurements of height, weight, blood pressure, and resting heart rate.^24^ A self-administered health and lifestyle questionnaire provided information about smoking habits, alcohol consumption, marital status, family history of disease, and educational level. Baseline physical fitness assessment included push-up capacity and treadmill exercise tolerance tests conducted per standardized protocols.^25^ For push-ups, the firefighter was instructed to begin push-ups in time with a metronome set at 80 beats per minute. Clinic staff counted the number of push-ups completed until the participant reached 80, missed 3 or more beats of the metronome, or stopped owing to exhaustion or other symptoms (dizziness, lightheadedness, chest pain, or shortness of breath). Numbers of push-ups were arbitrarily divided into 5 categories in increments of 10 push-ups for each category. Exercise tolerance tests were performed on a treadmill using a modified Bruce protocol until participants reached at least 85% of their maximal predicted heart rates, requested early termination, or experienced a clinical indication for early termination according to the American College of Sports Medicine Guidelines (maximum oxygen consumption [V̇o_2_max]).^26^

Occupational activity status was provided by the fire departments to the medical clinic, and the vital status of firefighters who were retired was verified through a registry of Indiana firefighter deaths constructed during an earlier mortality study.^27^

### Main Outcomes and Measures

The main outcome that we examined in this study was incident CVD-related events among participants. Cardiovascular disease–related events were defined as incident diagnosis of coronary artery disease or other major CVD event (eg, heart failure, sudden cardiac death). Participants were followed up from the date of enrollment (initial examination during the study period) until an outcome event or December 31, 2010, whichever came first. Cardiovascular disease events were verified clinically at return-to-work evaluations, fitness-for-duty examinations, or subsequent periodic examinations, all performed by the same clinic.

### Statistical Analysis

Continuous characteristics were presented as mean (SD), whereas categorical variables were shown as frequency or percentage. Covariates were compared among groups using the χ^2^ test of independence for categorical data and the *t* test or the analysis of variance test, as appropriate, for continuous data. The primary analyses were restricted to firefighters who completed a test of baseline push-up capacity. Incidence rate ratios (IRRs) were computed using the Poisson regression model with log-link function. Cox proportional hazards regression models were used to model the association of V̇o_2_max categories and push-up groups with the time to each outcome of interest from baseline, adjusting for age and body mass index (BMI) (calculated as weight in kilograms divided by height in meters squared); the resulting hazard ratios together with the corresponding 95% CIs and *P* values were reported. Kaplan-Meier estimates for the cumulative risk of CVD event were computed in the different push-up categories, compared using the log-rank test, and presented as a Figure. Statistical analyses were conducted using SAS software, version 9.3 (SAS Institute Inc); all tests conducted were 2-sided, with *P* < .05 indicating statistical significance. Final statistical analyses were completed on August 11, 2018.

**Figure.**
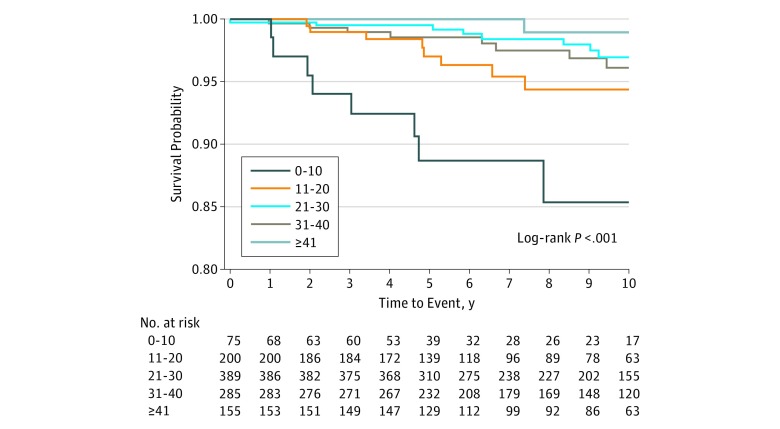
Kaplan-Meier Curves for the Cumulative Risk of Cardiovascular Disease Outcome in 5 Push-up Categories Push-up categories are by numbers of push-ups performed during baseline examination.

## Results

### Study Population

A total of 1562 eligible firefighters underwent baseline examination between February 2, 2000, and November 12, 2007, and were included in the initial retrospective cohort. Mean (SD) age at baseline for all participants was 39.6 (9.2) years and the mean (SD) BMI was 28.7 (4.3). Table 1 shows the baseline characteristics of the 1104 participants for whom push-up information was available stratified by push-up capacity at baseline examination. As summarized in Table 1, push-up capacity was found to be significantly inversely associated with most of the baseline risk factor variables that we examined (age, *P* < .001; BMI, *P* < .001; systolic blood pressure, *P* < .001; diastolic blood pressure, *P* < .001; total cholesterol level, *P* = .02; low-density lipoprotein cholesterol level, *P* = .04; triglycerides, *P* < .001; glucose level, *P* < .001; and smoking status, *P* < .001), although it was significantly positively associated with the estimated V̇o_2_max (*P* < .001). The baseline characteristics of firefighters without push-up data were similar and not statistically different from those who completed the push-up capacity evaluation on all markers of CVD risk.

**Table 1.** Descriptive Characteristics of Study Participants With Available Push-up Data Stratified by Number of Push-ups Performed During Baseline Examination

Variable	All Participants		0-10 Push-ups		11-20 Push-ups		21-30 Push-ups		31-40 Push-ups		≥41 Push-ups		*P* Value^a^
	No.	Mean (SD)	No.	Mean (SD)	No.	Mean (SD)	No.	Mean (SD)	No.	Mean (SD)	No.	Mean (SD)	
Age, y	1104	39.6 (9.2)	75	48.4 (10.1)	200	45.1 (8.6)	389	39.0 (8.3)	285	36.6 (8.0)	155	35.1 (7.1)	<.001
BMI	1101	28.7 (4.3)	75	33.1 (5.8)	200	30.3 (4.9)	388	28.7 (3.9)	285	27.4 (3.1)	155	26.8 (2.9)	<.001
Blood pressure, mm Hg													
SBP	1104	127.5 (12.0)	75	136.9 (17.9)	200	129.6 (12.1)	389	126.9 (11.8)	285	125.6 (10.3)	155	125.2 (9.4)	<.001
DBP	1104	85.7 (7.9)	75	89.4 (9.5)	200	86.5 (8.4)	389	85.9 (7.7)	285	84.6 (7.5)	155	84.0 (7.2)	<.001
Cholesterol level, mg/dL													
Total	1066	198.3 (38.1)	75	201.7 (43.0)	197	201.5 (35.6)	376	201.3 (39.8)	270	194.8 (37.2)	148	191.0 (34.9)	.02
HDL	1067	47.3 (23.1)	75	41.9 (10.6)	198	45.6 (15.1)	376	47.7 (34.6)	270	48.3 (12.4)	148	49.6 (11.2)	.13
LDL	1030	125.3 (42.0)	71	130.6 (33.3)	190	130.6 (70.8)	363	126.7 (32.3)	262	120.4 (31.4)	144	120.8 (31.3)	.04
Triglycerides	1066	145.2 (109.3)	75	167.9 (99.6)	197	162.2 (113.9)	376	150.9 (112.2)	270	134.5 (109.2)	148	116.1 (92.6)	<.001
Glucose level, mg/dL	1066	88.9 (16.4)	75	99.4 (24.3)	197	93.7 (22.9)	376	88.0 (13.9)	270	86.0 (12.4)	148	85.0 (8.4)	<.001
V̇o_2_max	1104	43.2 (6.3)	75	37.9 (6.5)	200	41.4 (6.0)	389	43.2 (6.2)	285	44.4 (5.7)	155	45.9 (5.4)	<.001
Race/ethnicity, No. (%)													
White	NA	964 (87.7)	NA	66 (88.0)	NA	170 (85.9)	NA	347 (89.2)	NA	245 (86.2)	NA	136 (88.3)	.95
African American	NA	118 (10.7)	NA	8 (10.7)	NA	25 (12.6)	NA	35 (9.0)	NA	34 (12.0)	NA	16 (10.4)	
Other	NA	18 (1.6)	NA	1 (1.3)	NA	3 (1.5)	NA	7 (1.8)	NA	5 (1.8)	NA	2 (1.3)	
Smoking status, No. (%)													
Nonsmoker	NA	617 (57.7)	NA	34 (45.3)	NA	82 (41.2)	NA	216 (57.4)	NA	182 (67.4)	NA	103 (69.1)	<.001
Previous smoker	NA	295 (27.6)	NA	23 (30.7)	NA	70 (35.2)	NA	102 (27.1)	NA	64 (23.7)	NA	36 (24.2)	
Current smoker	NA	157 (14.7)	NA	18 (24.0)	NA	47 (23.6)	NA	58 (15.4)	NA	24 (8.9)	NA	10 (6.7)	

Abbreviations: BMI, body mass index (calculated as weight in kilograms divided by height in meters squared); DBP, diastolic blood pressure, HDL, high-density lipoprotein; LDL, low-density lipoprotein; NA, not applicable; SBP, systolic blood pressure; V̇o_2_max, maximal oxygen consumption.

SI conversion: To convert total, HDL, and LDL cholesterol to mmol/L, multiply by 0.0259; triglycerides to mmol/L, multiply by 0.0113; and glucose to mmol/L, multiply by 0.0555.

^a^ *P* value based on an analysis of variance or χ^2^ test.

### Association of Push-ups and CVD-Related Outcomes

During the 10-year follow-up period, 37 CVD-related outcomes (1104 participants, 8601 person-years) occurred among firefighters with recorded push-up capacity data at baseline and were included in our analyses (Table 2). We observed significantly lower CVD IRRs in all groups with higher push-up capacity compared with the group with the lowest baseline push-up capacity. Participants able to complete more than 40 push-ups had a 96% reduction in incident CVD events compared with those completing fewer than 10 push-ups (IRR, 0.04; 95% CI, 0.01-0.36). In comparison, we observed similar crude IRRs of CVD outcomes when stratifying the cohort by estimated V̇o_2_max baseline.

**Table 2.** Statistical Analyses of Incidence and Rates of Cardiovascular Disease Outcome Stratified by Push-up Categories and Maximal Oxygen Consumption^a^

Category	Events (n = 37)	Rate, per 100 000 Person-years	IRR (95% CI)
Push-ups			
0-10	8	1757	1 [Reference]
11-20	9	625	0.36 (0.14-0.92)
21-30	9	288	0.16 (0.06-0.42)
31-40	10	433	0.25 (0.10-0.62)
≥41	1	79	0.04 (0.01-0.36)
Estimated V̇o_2_max, mL/kg per min			
≤35	3	904	1 [Reference]
36-42	15	385	0.43 (0.12-1.47)
43-49	8	327	0.36 (0.10-1.37)
50-56	10	641	0.71 (0.20-2.58)
≥57	1	273	0.30 (0.03-2.91)

Abbreviations: IRR, incidence rate ratio; V̇o_2_max, maximal oxygen consumption.

^a^ Cardiovascular disease outcome was defined as cardiovascular events including diagnoses of coronary artery disease or other major cardiovascular disease event.

Table 3 shows the results of the Cox regression models for the association of push-ups and V̇o_2_max groups with CVD outcomes. Even after adjusting for age and BMI, we observed an independent association of push-up capacity with CVD outcomes. Increased capacity was associated with a lower risk for CVD outcomes, with the comparison of the 21- to 30-push-ups group vs the 0- to 10-push-up group being statistically significant (hazard ratio, 0.25; 95% CI 0.08-0.76), although the other group comparisons did not reach statistical significance.

**Table 3.** Comparison Between Multiple Models of the Association of Maximal Oxygen Consumption or Push-up Categories With Cardiovascular Disease Outcome^a^

Model	HR (95% CI) Adjusted for Age^b^	*P* Value	HR (95% CI) Adjusted for Age and BMI^c^	*P* Value
**Model 1 (V̇o_2_max)**				
5 vs 1	0.52 (0.05-5.16)	.58	0.56 (0.05-5.90)	.63
4 vs 1	1.51 (0.40-5.76)	.54	1.60 (0.38-6.67)	.52
3 vs 1	0.75 (0.19-3.00)	.69	0.81 (0.18-3.71)	.78
2 vs 1	0.53 (0.15-1.85)	.32	0.56 (0.15-2.06)	.38
**Model 2 (Push-up Categories)**^b^				
5 vs 1	0.15 (0.02-1.29)	.08	0.14 (0.02-1.22)	.07
4 vs 1	0.60 (0.21-1.67)	.32	0.53 (0.17-1.66)	.28
3 vs 1	0.33 (0.12-0.90)	.03	0.31 (0.11-0.89)	.03
2 vs 1	0.47 (0.18-1.23)	.12	0.45 (0.17-1.20)	.11
**Model 3 (V̇o_2_max and Push-up Categories)**^b^				
V̇o_2_max				
5 vs 1	0.63 (0.06-6.38)	.69	0.54 (0.05-5.89)	.61
4 vs 1	2.09 (0.52-8.48)	.30	1.82 (0.41-8.10)	.43
3 vs 1	0.89 (0.22-3.66)	.87	0.74 (0.15-3.60)	.71
2 vs 1	0.64 (0.18-2.32)	.50	0.57 (0.15-2.23)	.42
Push-up categories				
5 vs 1	0.13 (0.01-1.14)	.07	0.11(0.01-1.07)	.06
4 vs 1	0.52 (0.17-1.54)	.23	0.46 (0.14-1.49)	.20
3 vs 1	0.27 (0.09-0.78)	.02	0.25 (0.08-0.76)	.01
2 vs 1	0.43(0.16-1.19)	.10	0.42 (0.15-1.15)	.09

Abbreviations: BMI, body mass index (calculated as weight in kilograms divided by height in meters squared); HR: hazard ratio; V̇o_2_max, maximal oxygen consumption.

^a^ Push-up categories are defined as follows: category 1, 0 to 10 push-ups; category 2, 11 to 20 push-ups; category 3, 21 to 30 push-ups; category 4, 31 to 40 push-ups; and category 5, 41 push-ups or more. Cardiovascular disease outcome was defined as cardiovascular events including diagnoses of coronary artery disease, or other major cardiovascular disease event and included 37 events per 8601 person-years among 1104 participants.

^b^ Adjusted for age using the Cox proportional hazards model.

^c^ Adjusted for age and BMI using the Cox proportional hazards model.

The Figure shows Kaplan-Meier curves for the cumulative risk of CVD outcome events for each category of baseline push-up capacities, with the 0- to 10-push-ups group having the greatest cumulative incidence of CVD outcome events. The Figure shows a 15% cumulative incidence of CVD events in the 0 to 10 push-ups group vs 5% or lower in the other groups.

## Discussion

This study found that push-up capacity was inversely associated with 10-year risk of CVD events among men aged 21 to 66 years. Thus, push-up capacity, a simple, no-cost measure, may provide a surrogate estimate of functional status among middle-aged men. Participants able to perform 11 or more push-ups at baseline had significantly reduced risk of subsequent CVD events. To our knowledge, this is the first study to report the inverse relationship between push-up capacity at baseline and subsequent CVD-related outcomes in an occupationally active male cohort.

Previous cross-sectional studies have incorporated push-ups in the assessment of muscular fitness and its correlation with cardiometabolic risk markers.^28,29^ In those studies, the authors found that a higher level of muscular strength was associated with lower cardiometabolic risk independent of cardiorespiratory fitness in the cohorts observed. Muscular strength has been shown to have an independent protective effect for all-cause mortality and hypertension in healthy males and is inversely associated with metabolic syndrome incidence and prevalence.^30^ However, most of those studies were either cross-sectional^29,31,32^ or conducted with adolescent participants.^28,29^ Our retrospective cohort study provides further insights into the association of greater fitness, specifically muscular strength, with CVD-related outcomes in an occupationally active cohort across 10 years of follow-up. Statistical adjustment for age and BMI suggested that some of the risk reduction seen with higher push-up categories was accounted for by these characteristics. There was also evidence that differences in established CVD risk factors (blood pressure, serum lipid levels, and smoking behavior) may explain much of the residual differences in outcome. Nonetheless, the results of this study support an inverse association between push-ups and CVD events among middle-aged men. In the present study, push-up capacity was more strongly associated with future CVD risk than was V̇o_2_max as estimated by submaximal stress tests. Previous studies have documented that submaximal stress tests can overestimate or underestimate CRF owing to the assumptions involved with extrapolating from submaximal to maximal results.^33,34^

The use of age and BMI in occupational settings as determinants of fitness for duty has been avoided owing to concerns related to the Americans with Disabilities Act.^35,36,37^ However, push-up capacity is a functional test. Many fire and police departments have neglected to provide periodic medical examinations or functional tests such as stress tests owing to cost concerns.^38,39^ The push-up examination requires no special equipment, is low cost or no cost, can easily be performed in almost any setting within 2 minutes, and provides an objective estimate of functional status. It is a quantitative measurement that is easily understood by both the clinician and the patient. The use of push-up capacity could assist clinicians in presenting objective information on CVD risk and in formulating physical activity and weight reduction prescriptions for patients. Fonarow et al^40^ suggested that the workplace is an important setting for promoting cardiovascular health; however, current workplace wellness programs often lack the appropriate physical activity assessment tools and the resources for exercise tolerance tests. Therefore, push-up capacity could potentially be an easily implemented tool and used as part of a comprehensive workplace wellness program.

Previous cross-sectional research in firefighters established that those with low CRF were significantly more likely to have abnormal results on maximal stress tests, worse CVD risk profiles, and a higher prevalence of metabolic syndrome, suggesting a higher future risk of CVD events.^21,41^ As a result, it is recommended that individuals with low CRF (<12 metabolic equivalents) receive cardiovascular risk reduction management.^21^ Again, a significant limitation of this recommendation is the requirement to perform costly and time-consuming maximal exercise stress tests. Most fire departments have been unable to adopt this recommendation for career firefighters, let alone for the volunteer firefighters who compose 70% of the fire service nationally.^42^ The use of push-up capacity has the potential to improve the feasibility and implementation of physical fitness assessment in workplace settings. Based on the current study, CVD risk factor reduction should be recommended, including lifestyle measures for those with low push-up capacity, especially those capable of 10 or fewer push-ups.

### Strengths and Limitations

The major strength of this study is the use of an objective surrogate measure of functional status, push-up capacity, from baseline physical examinations. Although push-up capacity may be influenced by technique and practice, the use of a standardized protocol for push-up performance by clinic staff would minimize the effect of technique on capacity. Previous studies have used self-reported physical activity questionnaires to assess duration, intensity, and frequency of physical activity; most of these categorized physical activity by total activity time.^2,15,17^ Thus, our study avoided participant recall bias and other well-known limitations of self-reported data. Our sample size and follow-up period of 10 years also provided sufficient data on CVD-related outcomes and exit events to observe significant associations. Another strength is the generalizability of the study cohort to other US firefighters. The cohort of Indiana career firefighters is similar with respect to age, race/ethnicity, and diet to other multistate cohorts.^43,44^ Our within-group analyses and comparisons and use of a statewide database of death certificates mitigated concerns for bias owing to a healthy worker effect or loss to follow-up.

The current study has several limitations. First, the study assessed the association between push-ups and CVD events. The results do not support push-up capacity as an independent predictor of CVD risk. Second, because the study cohort consisted of middle-aged, occupationally active men, the study results may not be generalizable to women, older or nonactive persons, other occupational groups, or unemployed persons. Further studies are needed to examine the association between push-up capacity and CVD incidence in a more general population of middle-aged men as well as among older individuals and women. The fitness evaluation was voluntary for the firefighter population used for this study; thus, the baseline measurements were missing information for approximately one-fourth of participants. However, the characteristics of participants with missing push-up information were similar to those with available push-up information. Nonetheless, this decreased the statistical power. In addition, given that the tests performed were not adjusted for multiple comparisons, some of the significant findings could be due to chance.

## Conclusions

In this 10-year longitudinal study, participants able to complete more than 40 push-ups were associated with a significant reduction in incident CVD event risk compared with those completing fewer than 10 push-ups, which may be explained by significant differences in recognized CVD risk factors at baseline among the groups. The findings suggest that being able to perform a greater number of push-ups at baseline is associated with a lower incidence of CVD events among active adult men. Thus, results from this study suggest that it is reasonable for clinicians to assess functional status during clinical evaluations by using basic questions regarding activity. Further research is warranted to determine the association of push-up capacity with CVD risk in the general population and the potential use of push-ups as a clinical assessment tool.
